# Selective attention - psi performance in children with learning disabilities

**DOI:** 10.1016/S1808-8694(15)30086-0

**Published:** 2015-10-19

**Authors:** Vera Lúcia Garcia, Liliane Desgualdo Pereira, Yotaka Fukuda

**Affiliations:** a Assistant professor in the Speech Therapy Department of the Bauru Dentistry College, Sao Paulo University; b Full professor in the Speech Therapy Department, Discipline of Hearing Disorders, Sao Paulo Federal University - Paulista School of Medicine; c Full professor in the Otorhinolaryngology Department, Sao Paulo Federal University - Paulista School of Medicine. Sao Paulo University / Sao Paulo Federal University - Paulista School of Medicine

**Keywords:** learning, hearing, child, dyslexia

## Abstract

Selective attention is essential for learning how to write and read. **Aim:** The objective of this study was to examine the process of selective auditory attention in children with learning disabilities. **Material and Method:** Group I included forty subjects aged between 9 years and six months and 10 years and eleven months, who had a low risk of altered hearing, language and learning development. Group II included 20 subjects aged between 9 years and five months and 11 years and ten months, who presented learning disabilities. A prospective study was done using the Pediatric Speech Intelligibility Test (PSI). **Result:** Right ear PSI with an ipsilateral competing message at speech/noise ratios of 0 and -10 was sufficient to differentiate Group I and Group II. Special attention should be given to the performance of Group II on the first tested ear, which may substantiate important signs of improvements in performance and rehabilitation. **Conclusion:** The PSI - MCI of the right ear at speech/noise ratios of 0 and -10 was appropriate to differentiate Groups I and II. There was an association with the group that presented learning disabilities: this group showed problems in selective attention.

## INTRODUCTION

Learning may be defined as a central nervous system process in which a varied amount of permanent changes is produced that affect function or behavior; these changes improve adaptation of an individual to its milieu as a response to an environmental action.^1^

There is no consensus on the identification of learning disabilities. This is due in part to the complexity of this phenomenon, evidenced by the absence of a single variable that could be identified as the primary source of learning disabilities.^2^ There is a consensus that in all of the definitions of learning disabilities there is a description of one or more altered language-related processes.^3^, ^4^ The term learning disabilities is a non-specific expression for a heterogeneous group of disorders that manifest as a significant difficulty to acquire and to use oral comprehension, speech, reading and writing abilities, reasoning and mathematical abilities.^5^ These disorders are intrinsic to each individual, possibly due to a central nervous system disorder, and may occur throughout life. Self-regulating behavior problems, lack of social perception and interaction difficulties may coexist with learning disabilities, although not in themselves learning problems. Furthermore, although learning disabilities may occur concomitantly with conditions such as sensory loss, mental retardation and severe emotional disorders or with extrinsic influences such as cultural differences and inadequate or insufficient instruction, learning disabilities are not a consequence of these conditions or influences.^5^

Attention is a multidimensional construct referring to a variety of relations between environmental stimuli and behavioral tasks and responses.^6^ Selective attention involves focusing on some mental activity to the detriment of others.^7^ In this case one or more stimuli produce relevant information, such as in tasks involving competing messages. In this situation, a subject is asked to hear some information and to ignore the remaining input, focusing his attention on the required stimulus and recovering only one of the possible pieces of information. Selective attention may also be used in dichotic listening tasks, during the binaural integration stage, where a subject is asked to recover both stimuli. Selective attention enables subjects to monitor specific significant auditory stimuli even when primary attention originates from another sense. It also enables subjects to react to a specific auditory stimulus and to ignore background noise.^8^, ^9^, ^10^ The figure-background, which is related to selective attention, is the ability to identify a primary message in the presence of competing sound.^11^ Selective attention is important for daily life activities such as reading in a noisy environment or learning new school material in a classroom where other competing stimuli are present. More than the amount of information that may be retained, selective attention requires individual control, which develops significantly between ages seven and ten years. Resistance to distraction by a competing stimulus remains constant with aging.^12^

It is clear that difficulties in extracting acoustic cues from auditory information and recognizing auditory patterns and/or short-term memory will influence the ability to focus on a task. Subjects with these problems find it difficult to process audition even in a silent environment.^13^

Memory is essential for every learning and adaptation process. Acquisition of a new behavior requires the possibility to compare what is perceived with what is already known.^1^ Neurocognitive mechanisms and processes are involved in auditory tasks, some of which deal specifically with auditory stimuli while others involve other functions, such as attention and long-term language representation.^3^, ^4^, ^14^

The Pediatric Speech Intelligibility (PSI) is an auditory processing test for selective attention;^15^ subjects must point to the corresponding figure when hearing a phrase presented together with a story. In this sense, process (selective attention) and ability (figure-background) may be taken as synonyms. Furthermore, the test task that subjects must perform to solve the problem of separately identifying overlapped and simultaneous information is dichotic or monochotic, depending on whether information reaches one ear (monaural) or both (binaural). Subjects perform dichotic tasks in the PSI test condition where they identify sentences with a contralateral competing message (CCM). The monochotic task is the PSI test condition involving an ipsilateral competing message (ICM). In Pediatric Speech Intelligibility with a contralateral competing message (PSI-CCM), subjects are asked to perform a binaural separation dichotic task in which selective attention separates information presented binaurally; this task requires figure-background auditory abilities. In Pediatric Speech Intelligibility with an ipsilateral competing message (PSI-ICM), subjects are asked to perform a monochotic tasks in which selective attention separates information presented monaurally; this task also requires figure-background auditory abilities. Such low redundancy information is presented as overlapped and simultaneous messages to the same ear.

Some authors that have used various auditory processing tests have underlined the association between auditory information processing and learning.^16^, ^17^, ^18^, ^19^, ^20^, ^21^, ^22^, ^23^, ^24^ In one paper, hearing-related selective attention processes were studied in 352 normal pre-school children, in children with learning disabilities and in children in which learning disabilities were suspected. Results showed that these processes were at risk in 90% of those children with learning disabilities.^25^

The aim of this trial was to investigate selective attention mechanisms and processes in children with and with no learning disabilities, to describe and to analyze their responses. Variables that were taken in to account were sex, the tested ear and the number/percentage of correct answers.

## MATERIAL AND METHODS

This trial was submitted to the Research Ethics Committee of the university in which the investigation was carried out according to National Health Council guidelines (resolution 196/96) and was approved (protocol number 1726/98).

The trial involved 60 subjects (36 male and 24 female) aged from 9 years and 5 months to 11 years and 10 months. The children were enrolled in the third and fourth years of basic education in the same school, and were divided into two groups. The control group (group I) was composed of 40 subjects (20 male and 20 female) aged from 9 years and 6 months to 10 years and 11 months. Children that had been diagnosed as having learning disabilities formed the learning disabilities group (group II), which was composed of 20 subjects (16 male and 4 female) aged from 9 years and 5 months to 11 years and 10 months.

Group I subjects were selected according to the following criteria:

1 Brazilian nationals native speakers of Brazilian Portuguese;
2 absence of a family history of auditory, development and learning disabilities; evidence of normal development and absence of a family history of congenital, otological or neurological diseases, as investigated through a family interview;
3 absence of evident signs of neurological disease in a clinical assessment that included the traditional neurological exam;^26^, ^27^
4 absence of evident signs of otological disease on otoscopy;
5 absence of hearing loss, confirmed through a basic audiological assessment that included pure tone audiometry, logoaudiometry and acoustic immitance testing;
6 absence of evident signs of a lower-than-average mental age, as identified by the Wechsler Intelligence Scale for Children (WISC)^28^;
7 absence of schooling difficulties according to teachers and pedagogic coordinators of the school in which the children were registered;
8 absence of altered articulated spoken language based on spontaneous conversation and an articulation test done by a speech therapist;
9 absence of written language difficulties as assessed by a dictation given by a speech therapist, and the analysis of notebooks and texts written by the students.

The neurological, otological, audiological exams in group I subjects were within normal limits. This group also had a normal intellectual coefficient, and standard oral and graphical performances.

Group II subjects were diagnosed as having learning disabilities,^5^, ^29^ and were selected according to the following criteria:

1 Brazilian nationals, speakers of Brazilian Portuguese;
2 absence of a family history of impaired neurological development, evidence of normal neurological development and absence of a family history of congenital or neurological diseases as shown in the family interview;
3 absence of evident signs of neurological disease in a clinical assessment that included the traditional neurological exam^26^, ^27^;
4 absence of evident signs of a lower-than-average mental age, as identified by the Wechsler Intelligence Scale for Children (WISC)^28^;
5 absence of evident signs of otological disease on otoscopy;
6 absence of hearing loss, confirmed through a basic audiological assessment that included pure tone audiometry, logoaudiometry and acoustic immitance testing;
7 presence of a school report showing learning difficulties, particularly of the graphic code, according to the teacher and the pedagogic coordinator of the school in which the child was registered;
8 presence of a diagnosis of learning disorder, with age-incompatible graphical production and learning, with at least a 2-year discrepancy between school performance and school level. Subjects with learning disabilities underperform substantially in reading and writing compared to the expected performance level for their age, schooling and intelligence level.

The normal auditory threshold criterion was the presence of auditory levels below 20dBNA (ANSI standards, 1969) at all assessed frequencies, namely, 250, 500, 1000, 2000, 3000, 4000, 6000 and 8000Hz. The normal criterion for the recorded speech recognition index was a value between 88 and 100%.^30^, ^31^ Tympanometry and measurement of the acoustic stapedial reflex threshold (ASRT) followed international standards.^32^

PSI test1^5^, ^33^, ^34^, ^35^ results were the object of this paper. PSI testing consisted of message identification with a CCM and an ICM in an acoustic booth. Test figures^15^ were presented for recognition and only then children were instructed to pay attention and point to the figures that corresponded to the sentence they had listened to while ignoring the competing message (story). The presentation intensity of a speech signal was 40dBNS, with mean tonal auditory thresholds at 500, 1000 and 2000Hz. The test was applied initially with a right ear CCM at a speech-in-noise ratio of 0 and -40, and then in the left ear under the same speech-in-noise condition. In a second stage the test was applied together with a right ear ICM at a speech-in-noise ratio of 0, -10 and -15, and then in the left ear under the same speech-in-noise condition. Although the PSI test was originally indicated for children up to age seven years,^33^, ^15^ we chose to apply the test in all of our sample, considering that the Synthetic Sentences Identification (SSI) test,^36^, ^37^, ^38^ which would be more appropriate for the age group of our sample, requires command of the graphic code. Application of this test in group II children, which presented difficulties in learning the graphic code, would have been impossible.

Pure tone audiometry was done using a Madsen Electronics Midmate 622 audiometer, a TDH-39P earphone and MX-41AR earmuffs, calibrated according to the ANSI-89 standard. Tympanometry and ASRT were done with an Interacoustics Az7r device (probe tone at 220Hz). PSI was done using a Midmate 622 audiometer, TDH-39P earphone and MX-41AR earmuffs, in an acoustic booth. The audiometer was coupled to a Sony D-171 CD player.

A number of correct responses was set for each test and each test stage for groups I and II. The percentage of correct answers was calculated for each PSI condition (CCM and ICM) in various speech-in-noise ratios. In group I the mean, median, mode, standard deviation, and upper and lower limits of correct answers were measured in male and female children for each test. Groups I and II were then compared in different test stages. The following statistical test were used for analysis:

1 the independent t test,^39^ to compare the percentage of correct answers to the PSI test in males and females for each ear and to compare the performance of groups I and II.
2 the paired t test,^39^ to compare individual responses when PSI test stimuli where presented through earphones to the right and left ears.

The significance level for the statistical tests was 5% (p ≤ 0.05).

## RESULTS

**Figure 1:**
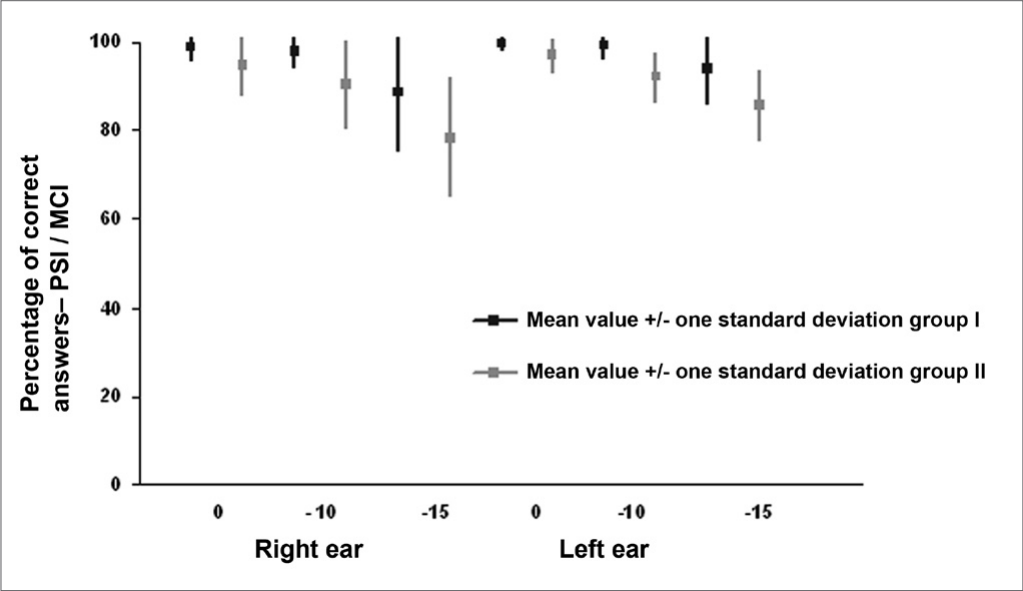
Mean (mean and standard deviation) percentage of correct answer values for right and left ears in groups I and II in the PSI with ipsilateral competing message (PSI-ICM) test at a speech-in-noise ratio of 0, - 10 and - 15.

Table 1 shows the results for group I, which were the mean values for the PSI-CCM and PSI-ICM tests and the statistical comparison for males and females, and right and left ears. Table 2 and Figure 1 show the mean values for groups I and II and the statistical analysis (independent t test) used for comparing the groups.

**Table 1:** Mean PSI-CCM and PSI-ICM values in Group I and the statistical results.

Subjects	PSI - CCM				PSI- ICM					
	RE	RE	LE	LE	RE	RE	RE	LE	LE	LE
	f/r = 0	f/r= -40	f/r = 0	f/r= -40	f/r = 0	f/r=10	f/r=-15	f/r = 0	f/r=-10	f/r=-15
Male	100,00	99,50	100,00	100,00	98,50	97,50	89,50	100,00	98,50	93,50
Female	100,00	100,00	100,00	100,00	99,50	99,00	83,00	99,50	99,50	92,50
Independent t test (M X F)	NA	0,324	NA	NA	0,305	0,223	0,119	0,330	0,305	0,695
Paired t test										
(RE X LE)	f/r 0 - NA		f/r - 40 - 0,323		f/r 0 - 0,183		f/r- 10 - 0,323		f/r-15 -0,002*	

PSI-CCM = PSI with contralateral competing message; PSI-ICM = PSI with ipsilateral competing message; s/n = speech-in-noise ratio; RE = right ear; LE = left ear; NA = not applicable

* statistically significant difference.

**Table 2:** Mean values in Group I and Group II, and the group comparison statistical results (independent t test).

Test	Group I	Group II	Statistical test
PSI-CCM RE s/n = 0	100,00	100,00	NA
PSI-CCM RE s/n = -40	99,75	100,00	04,84
PSI-CCM LE s/n = 0	100,00	99,5	0,330
PSI-CCM LE s/n = -40	100,00	100,00	NA
PSI-ICM RE s/n = 0	99,00	94,50	0,010*
PSI-ICM RE s/n = -10	98,25	93,50	0,050*
PSI-ICM RE s/n = -15	86,25	82,50	0,304
PSI-ICM LE s/n = 0	99,75	98,50	0,158
PSI-ICM LE s/n = -10	99,00	97,50	0,267
PSI-ICM LE s/n = -15	93,00	92,50	0,818

PSI-CCM = PSI with contralateral competing message; PSI-ICM = PSI with ipsilateral competing message; s/n = speech-in-noise ratio; RE = right ear; LE = left ear; NA = test not applied due to lack of variability

* statistically significant difference.

## DISCUSSION

Learning disorders are characterized by substantial underperformance in reading, writing and mathematics for age, schooling and intelligence.^29^ Estimates of the prevalence of learning disabilities range from 2 to 10%. Data suggest that 60 to 80% of dyslexic subjects - a specific type of learning difficulty for reading - are male.^29^ A Brazilian study showed that of 297 children diagnosed as dyslexic in a multidisciplinary evaluation, 78.45% were male and 21.55% were female.40 In this study, group II contained 16 male children (80%) and 4 female children (20%), similar to findings in the literature.^40^ Some authors have shown that many children with learning disabilities present auditory processing disorders,41-44 with a predominance in males; estimates suggest a male/female 8 to 1 ratio. ^41^

The statistical analysis for comparison of age differences in groups I and II showed that the mean age in group I was 121.78 months and the mean age in group II was 122.50 months, which was not statistically significant. This statistical comparison was made as differently aged groups are in different developmental neurological and maturation phases,^45^ which precludes an adequate comparison of performance between subjects. Brain myelination occurs at different rates in each region; the brainstem tracts are myelinated before subcortical regions of the brain.46 In human beings, brainstem audiometry shows adult myelination rates at about age 2 years; on the other hand, the mean, long latency and the P300 do not reach adult levels until preadolescence or adolescence.^46^, ^47^

Averages - the mean, median, mode and the standard deviation - and upper and lower limits were calculated for each of the tests applied to group I subjects. The statistical analysis showed that there were no significant differences between male and female results in the PSI-CCM test at speech-in-noise ratios of 0 and -40, and in the PSI-ICM test at speech-in-noise ratios of 0, -10 and -15 (Table 1). The authors that created this test also found no statistically significant difference between males and females during the PSI test standardization.48 These authors assessed 24 children (14 male and 10 female) aged from 3 years and 4 months to 9 years and found no sex-related performance difference. Our results confirm that the variable sex does not interfere with individual responses to the PSI test.

There was no statistically significant difference between individual right and left ear performance in the PSI-CCM test at speech-in-noise ratios of 0 and -40 and in the PSI-ICM test at speech-in-noise ratios of 0 and -10 (Table 1 and Figure 1). There was a statistically significant difference between right ear and left ear results at a speech-in-noise ratio of -15, where right ear responses - the first ear that was tested at this speech-in-noise ratio - were lower compared to left ear responses. (Table 1 and Figure 1). In a monochotic speech recognition in noise test, some authors have found that the second ear that was tested performed better, probably due to learning of test conditions, according to these authors.^31^, ^49^ Although auditory closure is the required ability for speech processing in noise,^9^, ^50^ and the figure-background ability is needed for PSI-CCM and PSI-ICM testing, both tests demand selective attention and task learning abilities. In our study task-learning appears to have favored the performance of the second ear that was tested (left ear) compared to the first ear that was tested (right ear).

Averages - the mean, median, mode and the standard deviation - and upper and lower limits were calculated for each of the tests applied to group II subjects. The variable sex was not compared as group II consisted of 16 males and 4 females. There was no statistically significant difference between right and left ear results in the PSI-CCM test at a speech-in-noise ratio of 0 and -40. There was no statistically significant performance difference between right and left ears in the PSI-ICM test at a speech-in-noise ratio of -10 (Figure 1). There was a statistically significant difference between right and left ears in the PSI-ICM test at a speech-in-noise ratio of 0 and -10, where right ear responses were lower compared to left ear responses (Figure 1). The right ear was assessed first and the left ear was assessed next at each speech-in-noise ratio. Similar to group I, it appears that learning was a significant factor in a monochotic speech recognition in noise test in group II, as has been pointed out by some authors.^9^, ^50^ A word of caution, however, is that in group I there was a difference between right and left ears only at the -15 ratio - a difficult to listen speech-in-noise ratio - where right ears had less errors. In group II, there was also a difference between right and left ears at a speech-in-noise ratio of 0 - a slightly distorted listening condition - besides the speech-in-noise ratio of -10, which suggests that in this task group II subjects required more items and/or acoustic cues to process auditory information, even under favorable listening conditions. The performance difference between right and left ears at a speech-in-noise ratio of 0 should be measured and taken into account when assessing children with learning disabilities, as this response was only found in group II of our study, and appears to characterize the auditory perception strategies that these children employed in this task. This finding may be useful to guide the rehabilitation process with greater precision. Children with altered auditory processing usually require more items to learn a task, and make more errors during the learning process. This has been called perceptual learning, and suggests that the most relevant acoustic characteristics of speech should be made more audible.^51^

There was no statistically significant difference between groups I and II in the PSI-CCM test (Table 2). In this trial, PSI-CCM was unable to differentiate both groups; there was no association between PSI-CCM test results and the group with learning disabilities. In this study the PSI-ICM test was unable to differentiate both groups in the right ear at a speech-in-noise ratio of -15 and in the left ear at speech-in-noise ratios of 0, -10 and -15; there was no association between PSI-ICM results and the diagnosed with learning disability group (Table 2 and Figure 1). The PSI-ICM test was able to differentiate groups I and II in right ear testing at a speech-in-noise ratio of 0 and -10; there was an association between PSI-ICM results and the learning disability group (Figure 1). Although some authors^23^, ^34^ have considered the PSI test efficient for diagnosing learning disabilities in children up to age 7 years, the efficiency of this test was confirmed only for the ICM condition and in some of the speech-in-noise ratios (0 and -10) in children up to age 11 years, which was our sample. Special attention should be given in the analysis of results to first tested ear responses (right ear), as performance differences between groups were the right ear responses at speech-in-noise ratios of 0 and -10. The figure-background ability is affected in group II subjects; lack of this ability in the school setting, which usually is noisy, may be related to some of the difficulties these children face. Difficulties in assimilating the content of what is taught may be due to not being able to interpret commands given in noise, particularly if there is an associated memory disorder. Selective attention processes in these subjects are compromised when there are other auditory stimuli. These children find it difficult, for instance, to understand what the teacher is saying; noise-induced stress may be added to this situation.^52^ These symptoms usually lead to distraction and other behaviors that are poorly accepted in the school environment.

Given the performance differences found in groups I and II, rehabilitation of these subjects should focus on metacognitive strategies and on improving the signal-to-noise ratio. Speakers and listeners could be placed closer to each other and the originators of noise should be kept further away; otherwise frequency modulation (FM) systems might be used.

## CONCLUSION

Our results show that there were no statistically significant differences in the comparison between males and females. There was a statistically significant difference between right and left ear results at a speech-in-noise ratio of -15 in the PSI ICM test. Right ear (first tested ear) responses at this speech-in-noise ratio were lower than left ear (second tested ear) responses at the same speech-in-noise ratio, probably suggesting a task-learning process. PSI-ICM testing was able to differentiate both groups (right ear) at speech-in-noise ratios of 0 and -10. There was an association between PSI-ICM results and the learning disabilities group, where selective attention processes were seen in the task; this finding indicates which aspects the audiologist should approach in rehabilitation.
